# Correction: Temporal information loss in the macaque early visual system

**DOI:** 10.1371/journal.pbio.3001752

**Published:** 2022-08-04

**Authors:** Gregory D. Horwitz

In the subsection SNR: LGN Populations within the Methods, the three equations immediately following Eq 11 should not have a superscript ^-1^. The section should read as follows:

The net signal was therefore *w*^*T*^*μ*, and the net noise was $\sqrt{w^{T}\sum_{pop}w}$ [135], leading to the SNR $\frac{w^{T}\mu }{\sqrt{w^{T}\sum_{pop}W}}$.

Signal and noise were pooled across two identical and weakly anticorrelated (r = −0.05 [87]) mosaics of neurons (e.g., representing ON and OFF neurons) and from a second, independent eye. The final scale factor was therefore

$$Population\;scale\;factor=\frac{4w^{T}\mu }{\sqrt{4.2w^{T}\sum_{pop}w}}$$ (12)
