# Supplementary figures and images for: Temporal information loss in the macaque early visual system
Source: PLoS Biol. 2020 Jan 23;18(1):e3000570. doi: 10.1371/journal.pbio.3000570 (PMC6977937; doi:10.1371/journal.pbio.3000570)

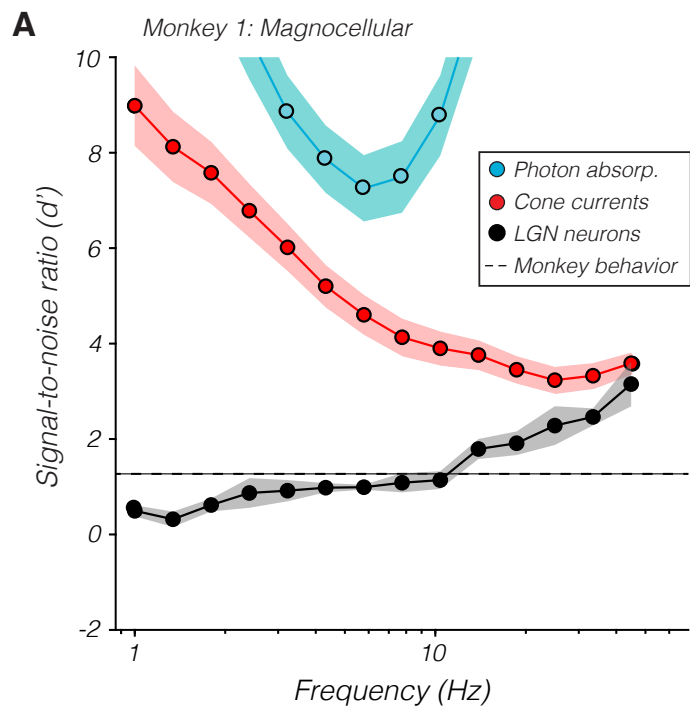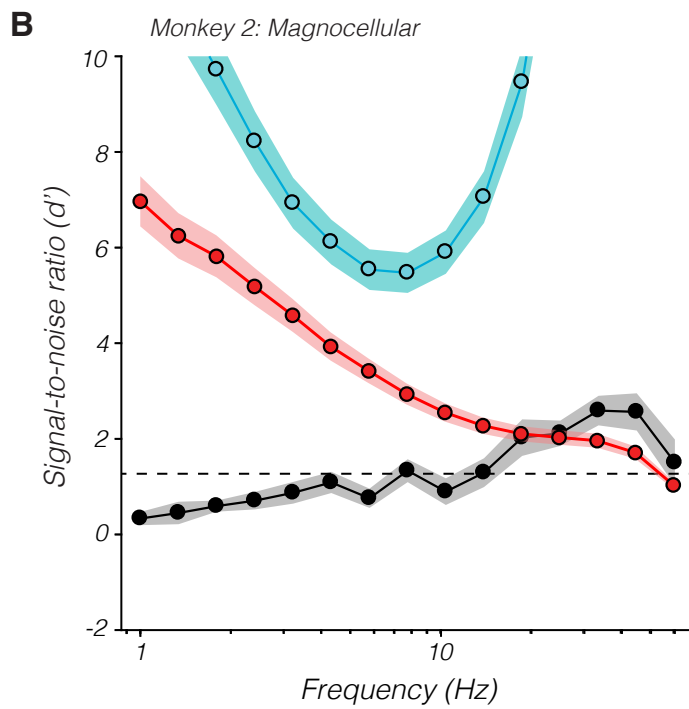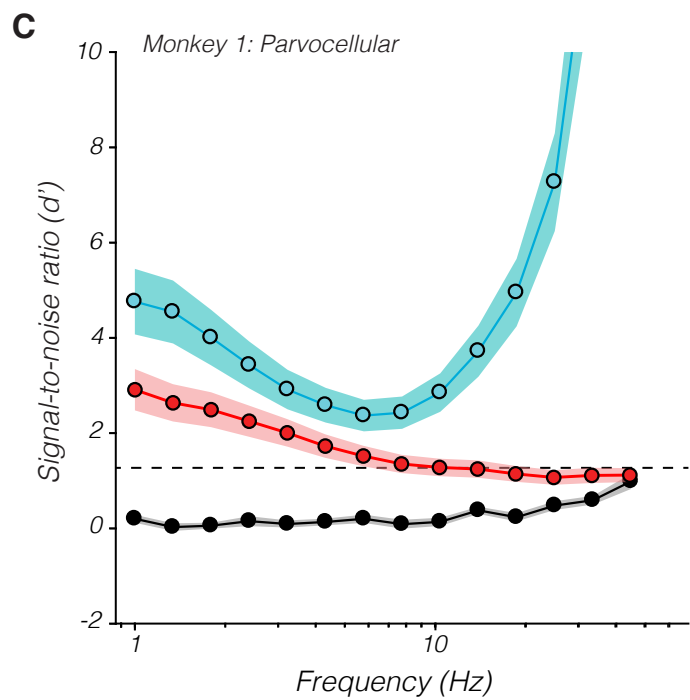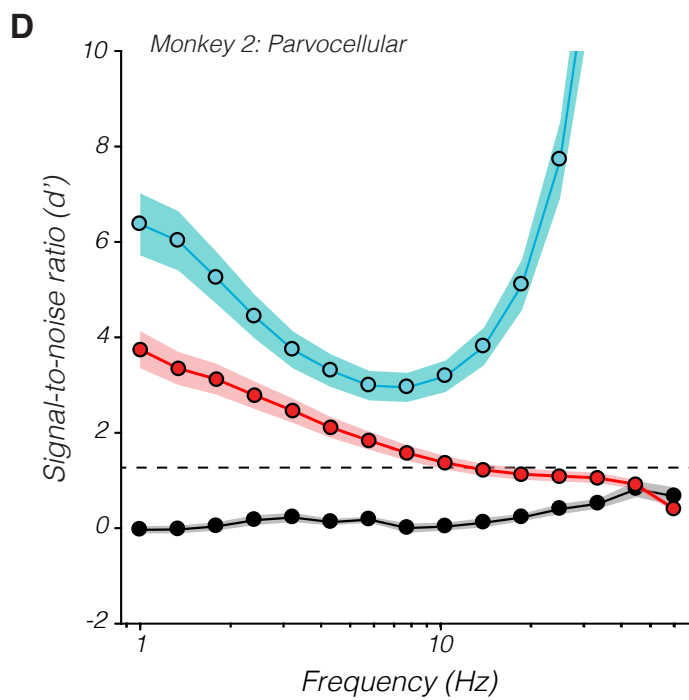

Supplement: S1 Fig — Signal-to-noise ratios (d′ values) of individual LGN neurons (black), currents in cones within their RFs (red), and photon absorptions in the same cones (cyan). Points are means and shaded bands are ±1 SEM. Dashed line indicates the signal-to-noise ratio assumed at the level of behavior on the basis of performance in a two-alternative, forced-choice contrast detection task (d′ = 1.27 or 82% correct). This analysis is identical to the analysis in Fig 5 of the main text except that the spatial integration window of the ideal observers was constrained to a single monocular LGN RF. This analysis does not require assumptions about pools of LGN neurons or interneuronal correlations. Notice that the relative signal-to-noise ratio is similar in small spatial integration windows (this figure) and windows that were, on average, 74 times larger (Fig 5). The mean signal-to-noise ratio across individual LGN neurons (black traces) also appears in Fig 4 of the main text. Data are available at https://github.com/horwitzlab/LGN-temporal-contrast-sensitivity/blob/master/DataByFigure.xlsx. LGN, lateral geniculate nucleus; RF, receptive field. (PDF) [file pbio.3000570.s001.pdf]

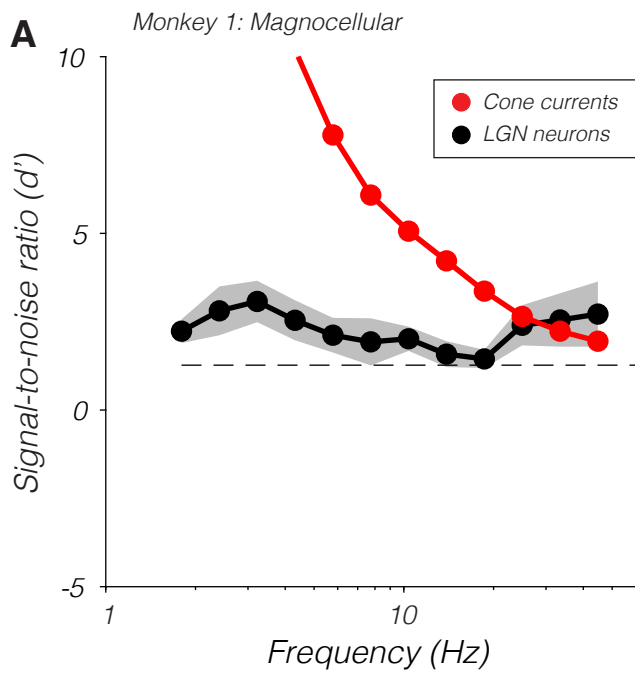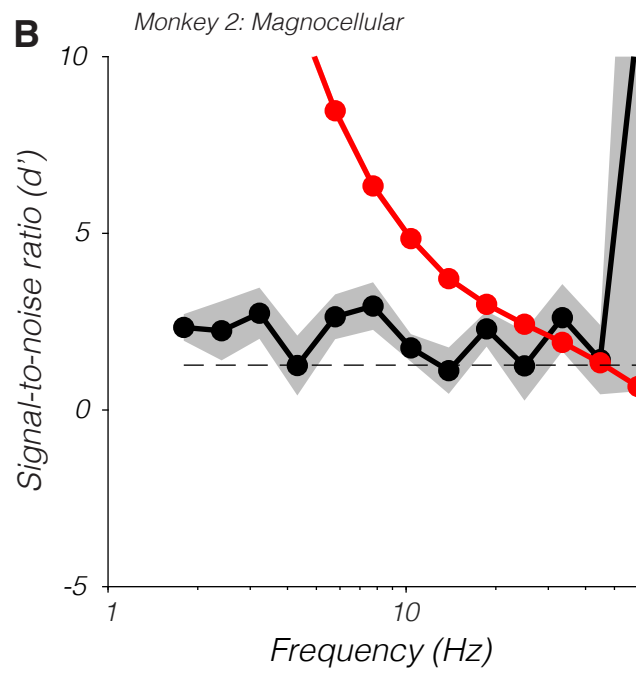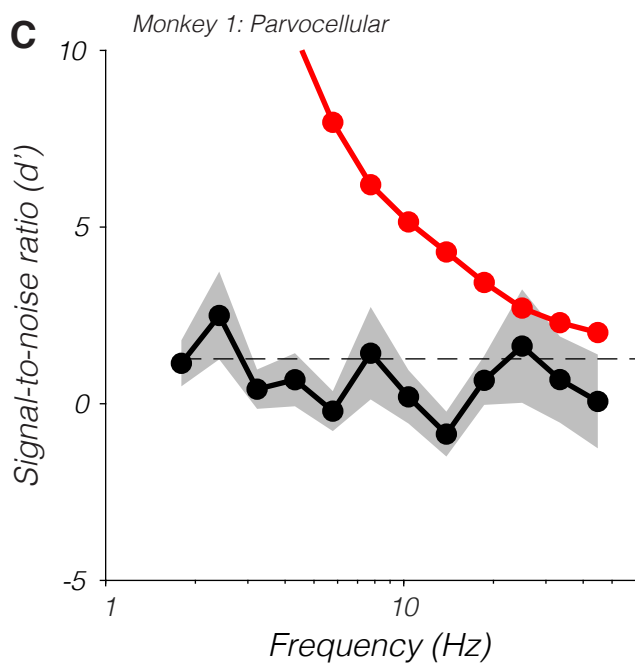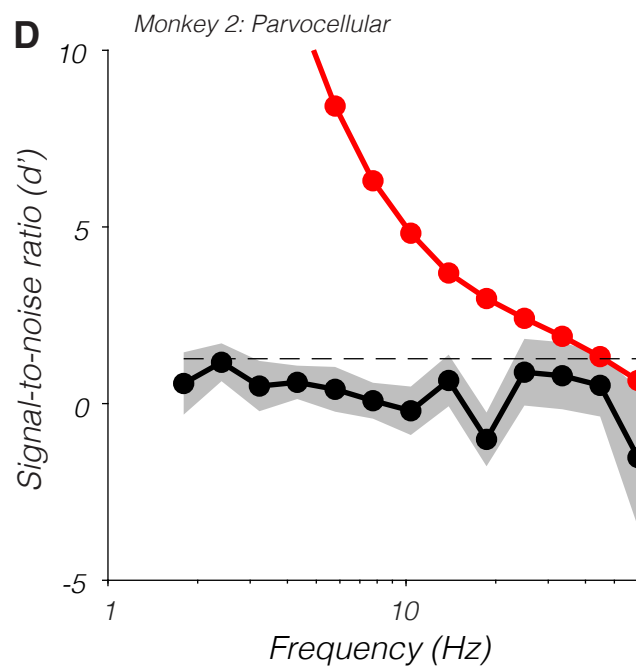

Supplement: S2 Fig — Signal-to-noise ratios (population d′ values, see Methods) calculated from single cycles of LGN neuronal responses (black) and simulated cone currents (red) as a function of temporal frequency. Points are means and shaded bands are ±1 SEM. Dashed line indicates the signal-to-noise ratio assumed at the level of behavior (d′ = 1.27 or 82% accuracy). Reducing the temporal integration window to include a single stimulus cycle resulted in signal-to-noise ratios being roughly constant across temporal frequency. (A) Magnocellular data from monkey 1. (B) Magnocellular data from monkey 2. (C) Parvocellular data from monkey 1. (D) Parvocellular data from monkey 2. Data are available at https://github.com/horwitzlab/LGN-temporal-contrast-sensitivity/blob/master/DataByFigure.xlsx. LGN, lateral geniculate nucleus. (PDF) [file pbio.3000570.s002.pdf]

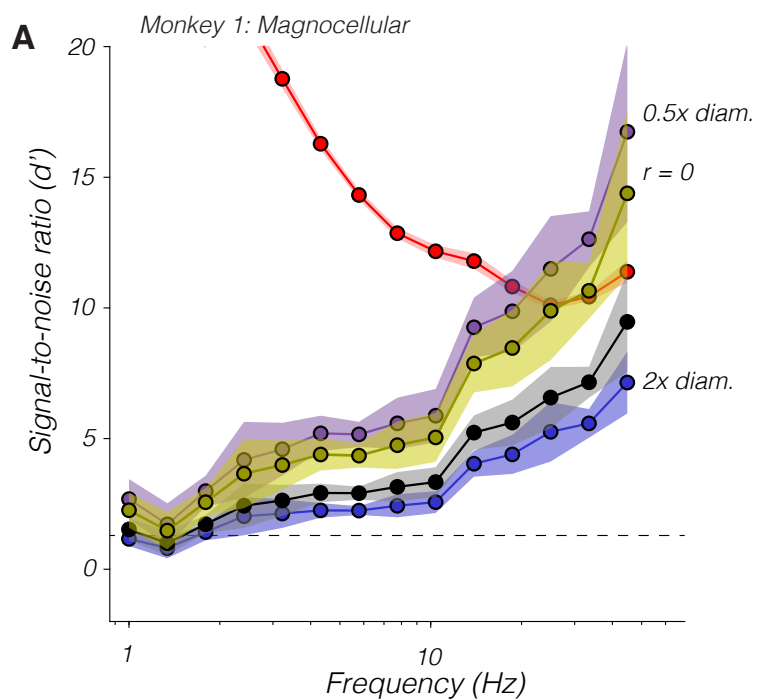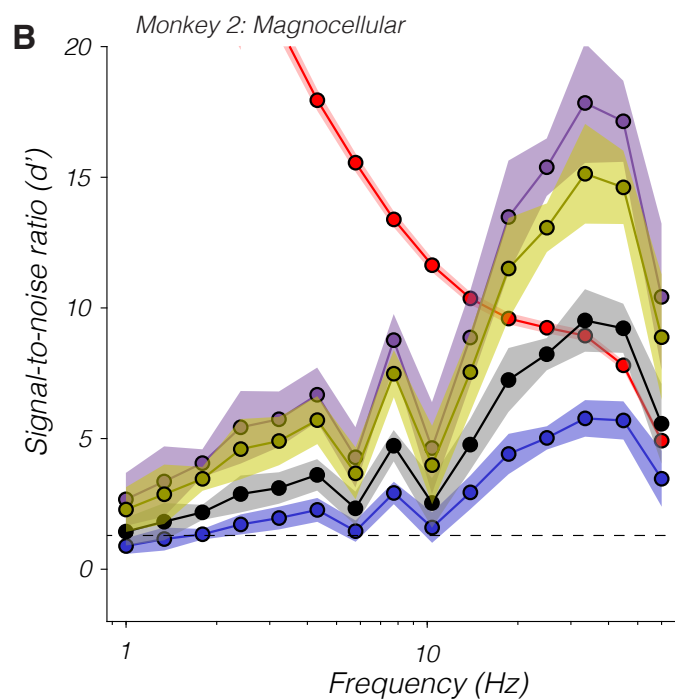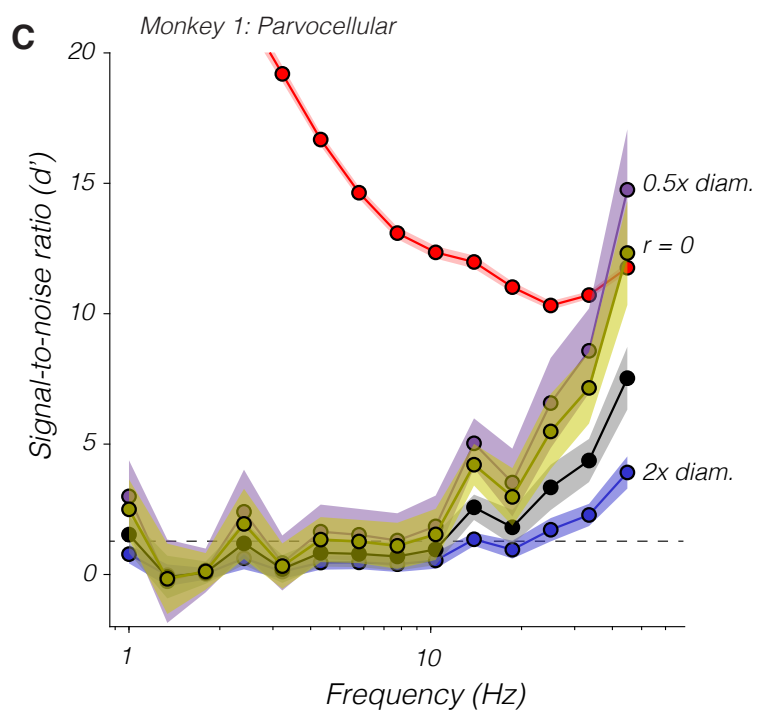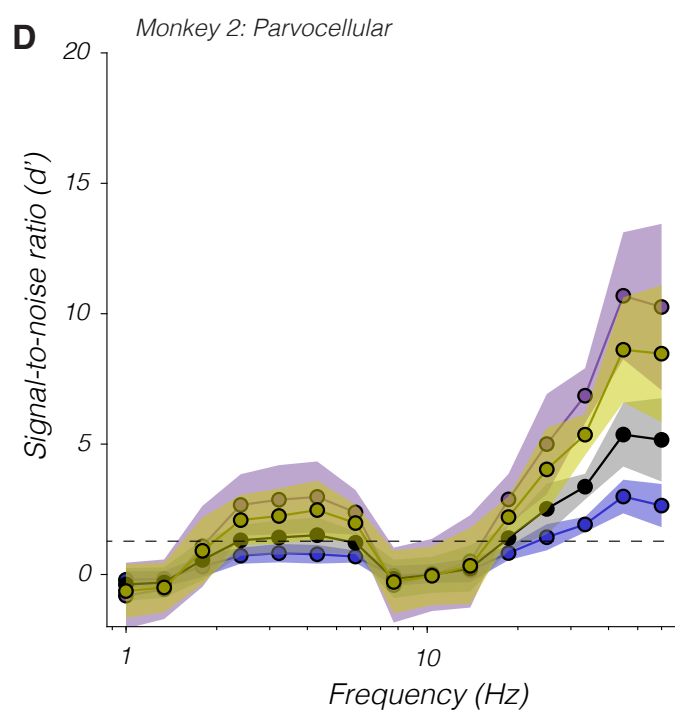

Supplement: S3 Fig — Signal-to-noise ratios (d′ values) of cone currents (red) and LGN populations under several pooling models. Conventions are identical to Fig 5 of the main text. LGN population scale factors were computed as described in the main text (black), in the absence of within-mosaic interneuronal correlations (yellow), after doubling RF diameter (blue), or after halving RF diameter (purple). All of these manipulations affect population scale factors and therefore scale the d′ curves. (A) Magnocellular data from monkey 1. (B) Magnocellular data from monkey 2. (C) Parvocellular data from monkey 1. (D) Parvocellular data from monkey 2. Data are available at https://github.com/horwitzlab/LGN-temporal-contrast-sensitivity/blob/master/DataByFigure.xlsx. LGN, lateral geniculate nucleus. RF, receptive field. (PDF) [file pbio.3000570.s003.pdf]

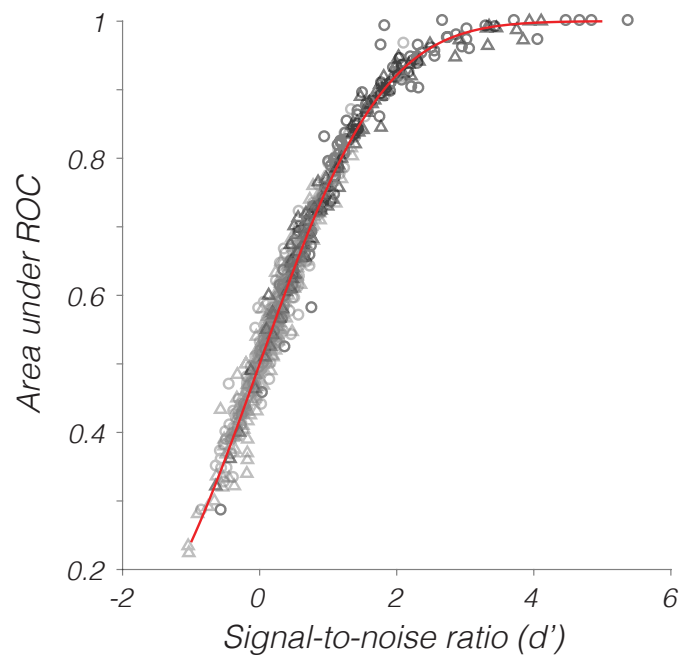

Supplement: S4 Fig — ROC curves were computed from dM2 values (see Methods, Eq 9). Each symbol represents the data from a single LGN neuron and a single temporal frequency. Data from parvocellular (gray) and magnocellular (black) neurons are shown from monkey 1 (circles) and monkey 2 (triangles). The red curve is the prediction from Gaussian signal and noise distributions with identical SD. Data are available at https://github.com/horwitzlab/LGN-temporal-contrast-sensitivity/blob/master/DataByFigure.xlsx. LGN, lateral geniculate nucleus; ROC, receiver operating characteristic; SD, standard deviation. (PDF) [file pbio.3000570.s004.pdf]

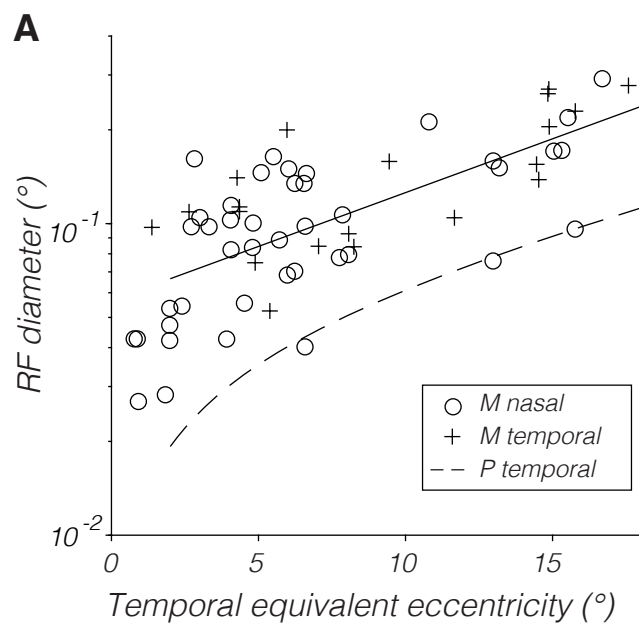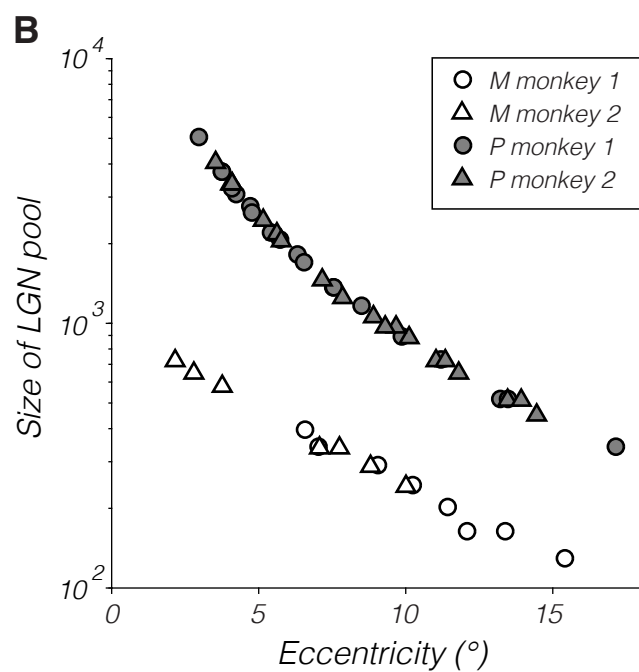

Supplement: S5 Fig — (A) Magnocellular and parvocellular RF diameter (2 SDs of a Gaussian fit) as a function of temporal equivalent retinal eccentricity. Points are RFs in the temporal (+) and nasal (circles) retinae from Derrington and Lennie [53]. The solid line is a least-squares fit. Parvocellular RF sizes were estimated from the model proposed by Watson [81] and shifted down by a factor of 0.8 to account for the smaller RF sizes of macaques [82] (dashed curve). (B) Number of LGN neurons assumed in each ideal observer pool as a function of eccentricity. Pool sizes were calculated on the basis of RF size, the assumption of a hexagonal RF lattice, and the retinal size of the stimulus (a Gabor with 0.15° SD envelope, truncated at ±2 SDs). Open circles represent magnocellular neurons and closed symbols represent parvocellular neurons. Circles and triangles represent neurons from monkeys 1 and 2, respectively. Data are available at https://github.com/horwitzlab/LGN-temporal-contrast-sensitivity/blob/master/DataByFigure.xlsx. LGN, lateral geniculate nucleus; RF, receptive field; SD, standard deviation. (PDF) [file pbio.3000570.s005.pdf]
