# Supplementary material for: Temporal information loss in the macaque early visual system
Source: PLoS Biol. 2020 Jan 23;18(1):e3000570. doi: 10.1371/journal.pbio.3000570 (PMC6977937; doi:10.1371/journal.pbio.3000570)
Supplement: Attachment — Submitted filename: ReviewerFigure1.pdf [file pbio.3000570.s006.pdf]

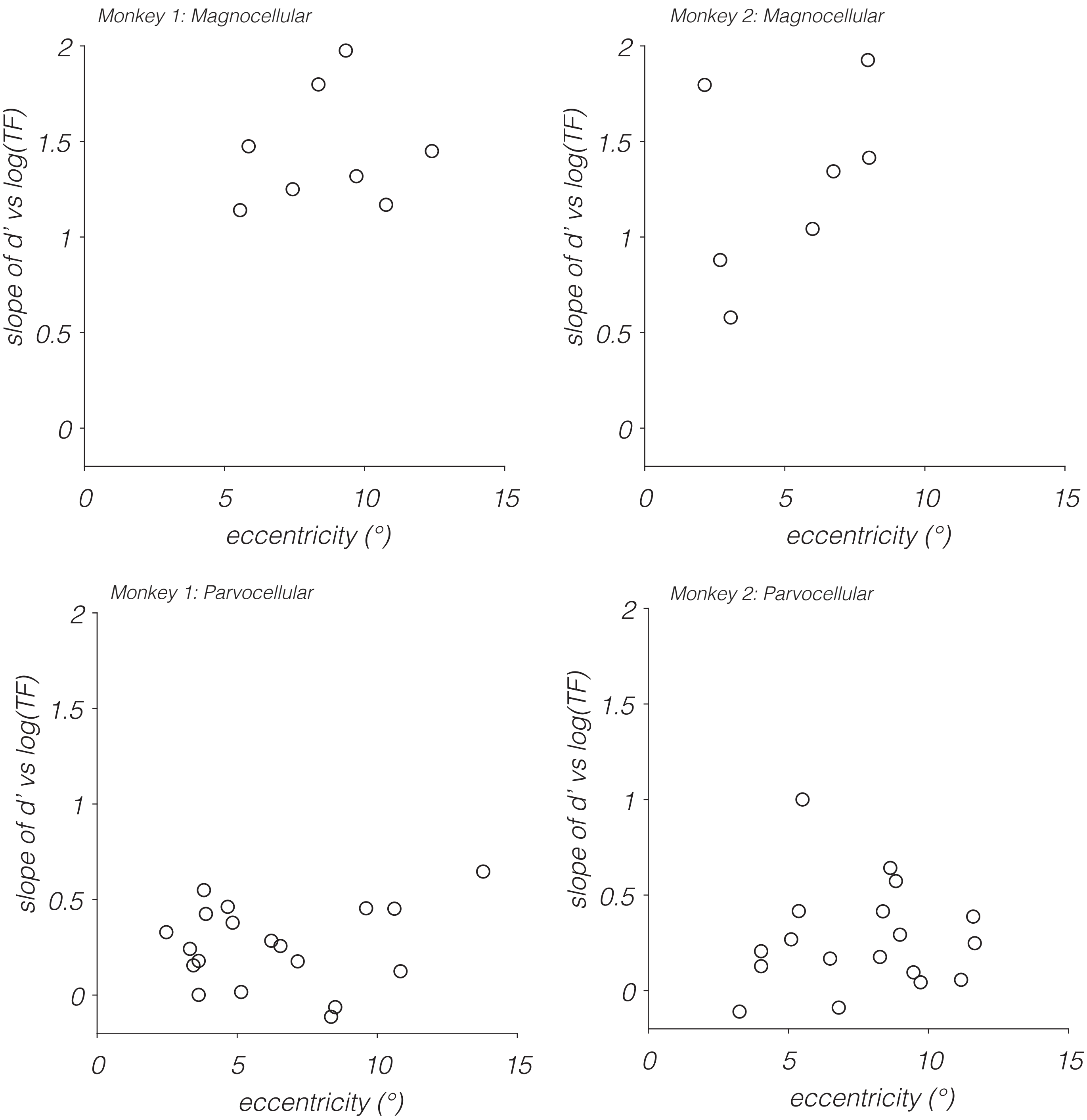

Reviewer Figure 1. d' of each LGN neuron was regressed on $\log_{10}$ temporal frequency . The fitted slopes are plotted here as a function of RF eccentricity. A specialization for high-temporal frequencies at large eccentricities might manifest as an increase in slope with eccentricity. While there are hints of such a relationship in the magnocellular neurons from monkey 2 and parvocellular neurons from monkey 1, none of these relationships attained statistical significance.
