# Supplementary material for: Temporal information loss in the macaque early visual system
Source: PLoS Biol. 2020 Jan 23;18(1):e3000570. doi: 10.1371/journal.pbio.3000570 (PMC6977937; doi:10.1371/journal.pbio.3000570)
Supplement: Attachment — Submitted filename: Response to Reviewers.docx [file pbio.3000570.s007.docx]

Attached please find a new submission based on the previously reviewed and rejected manuscript "Temporal information loss in the macaque visual system" [PBIOLOGY-D-19-01326R1].

------------------------------------------------------------------------

Reviewer remarks:

Reviewer #1: Review of PBIOLOGY-D-19-01326R1, ‘Temporal information loss in the macaque visual system’.

Note to Author: consider including line numbers in future submissions; in Comments (below), P = page, pp = paragraph.

This is an interesting study aimed at understanding visual perceptual limits. While it is known that peripheral sensory neurons may encode stimuli too weak to be perceived, where in the signaling pathway the underlying loss(es) of information occur is not known in detail for any sensory modality. The author of this manuscript asked where along the signaling pathway from photoreceptors to the level of visual perception visual information pertaining to a Gabor stimulus at threshold is lost. They compared discrimination sensitivity at two signaling stages within the photoreceptors (photon absorptions and electrical currents), one in the LGN, and at the behavioral level, using awake macaque monkeys in a two-alternative-forced-choice discrimination paradigm. Visual sensitivity in photoreceptors was modeled using parameters obtained from the literature. Sensitivity at the level of the LGN was computed from spike responses obtained with single unit recordings in vivo. Behavioral

sensitivity was obtained through established monkey psychophysics methods. To compare sensitivity at each stage, the author converted results at each level into a single ‘currency’: signal to noise ratio (SNR). Since SNR is a direct measure of discrimination sensitivity, this is both valid and useful.

The author reports that loss of visual information is temporal frequency-dependent: after phototransduction (which accounts for the greatest overall loss) high temporal frequencies are lost primarily after the LGN, supposedly through inefficient signaling with temporally redundant LGN spikes, whereas low temporal frequencies are lost primarily between the cones and the LGN. The author has made an impressive effort connecting and converting measures and methodology from previous studies.

Although the result are interesting, I have the following major concerns pertaining to the role of spatiotemporal integration, changes in which will move arounbd SNR and therefore directly impact the main claims, but the choices made and sensitivity of the results to it are insufficiently substantiated in this study. As is, substantial uncertainty remains over exactly where within the assessed signaling pathway information is lost, and how much.

Major concerns

1. Much of the interpretation hangs on the correctness of assumptions about spatial and temporal integration. As explained by the authors, integrating multiple neurons’ signals evoked by the same stimulus increases SNR in a manner that is known and predictable but dependent on signal and noise correlations, which are not necessarily known. Model assumptions about the neuron population sizes at different stages are based on earlier studies, referenced in the manuscript, but too opaque to be readily verified and interpreted by the reader. Because so much of the quantitative aspect of the comparison of SNR at subsequent stages hangs on the size of the neuronal pool assumed to contribute, neuronal pool size must be made more explicit, and the dependence of the main results on pools size shown in additional analyses. Without this data, accuracy of the main claims and their sensitivity to model assumptions cannot be independently verified.

All three reviewers expressed concern about the sizes of neuronal pools used in the LGN ideal observer model. To address this concern, the new submission includes a new figure showing pool sizes (Figure 10B), results from analyses in which these pool sizes are changed (Supporting Figure 3), links to Matlab code and data on which the pool sizes are based, and links to Matlab code that allows pool size to be changed.

Inefficient integration is one way that information is lost in the visual system. Importantly, the three ideal observers used in this study (photon absorptions, cone currents, and LGN responses) used the same spatiotemporal integration window, and their relative sensitivity is robust to changes in this window. Photon absorption noise is uncorrelated in time and space, so decreasing the integration window reduces the signal-to-noise ratio proportionally to the square root of stimulus contrast omitted from the window. The same logic holds approximately for the other two ideal observers: Cone current noise is uncorrelated in space and, for a given stimulus, has nearly equal power over the temporal frequencies considered by the ideal observer (for this observer, the variance of pooled cone currents was calculated in the Fourier domain; see response to Reviewer 2). LGN responses are weakly correlated in time and space (as evidenced by data from the current study, Greschner et al. 2011, and Jiang et al., 2015). Reducing the integration window thus reduces the sensitivity of each of these three ideal observers similarly.

To illustrate this point, I repeated a key analysis (Figure 5 in the manuscript), restricting spatial integration to individual, monocular LGN receptive fields. Results are shown in Supporting Figure 1. In this analysis, there is no pooling of LGN neurons so assumptions about pool sizes and interneuronal correlations are moot. The basic results are maintained: low frequency information is lost between the cones and the LGN, and high frequency information is lost within the cones and downstream of the LGN.

2. Similarly, temporal integration influences SNR in a frequency-dependent manner. Methods, P17, pp1, states that for the photon absorptions, ‘Poisson counts were weighted over space and time using a spatiotemporal weighting function that was identical to the stimulus’. It is not obvious why this was done, rather than using something more similar to the published cone impulse response.

The photon absorption ideal observer was inadequately explained. What I meant to say was that the ideal observer of photon absorption used the optimal weighting function, which happens to be identical to the stimulus. This coincidence is because photon absorptions are instantaneous, local, and independent across space and time.

Using the cone impulse response (which I interpret as meaning using a spatiotemporal weighting function that is equal to convolution of the stimulus with the cone impulse response) is hard to justify for this ideal observer. The cone impulse response is wide because phototransduction is slow. Phototransduction is downstream of the signal to which the ideal observer of photon absorptions has access.

To vary temporal frequency parametrically, long stimulus durations were unavoidable (brief stimuli distributed power across broad ranges of temporal frequency).

In response to a suggestion made by Reviewer 2, I reanalyzed the data, restricting ideal observers' integration to a single stimulus cycle. Under these conditions, signal-to-noise ratio in the LGN is largely independent of temporal frequency (see Supporting Figure 2). This result is an expected consequence of the increase in signal-to-noise ratio with temporal frequency in fixed integration windows and the decrease in stimulus period with temporal frequency.

What were the temporal integration time courses for subsequent neuronal stages? Some of the dependence of SNR and firing rate on integration time is shown in Figure 8, but how these results play into the overall results is not clear.

I am unsure how to interpret this question. Three stages downstream of photon absorptions were analyzed. The first is cone currents. I assume that each cone has a temporal integration time course that is given by the temporal impulse response estimated by Anguerya and Rieke (2013), which is similar to other estimates (e.g. Cao et al. 2014, Baudin et al. 2019). The second stage is the LGN. I did not make any assumptions about the temporal integration times of LGN neurons, I simply measured spiking responses. (That having been said, the linear model of Benardete and Kaplan (1997), which does assume an explicit integration time, does an excellent job of predicting the average responses of the parvocellular neurons I studied. I am happy to share these simulations if the reviewers think that this would be a useful addition.) The third stage is behavior. The temporal integration time of the monkey, behaviorally, is unknown but is probably close to that of humans (~40–100 ms) and stimulus-dependent in an intriguing way that the current results may help to explain. All ideal observers, being ideal, integrated over the entire stimulus duration.

Other comments

3. Results P3, pp3, That a ‘parvocellular neuron, with a nearby RF, responded similarly but relatively weakly (Figure 2B)’ is difficult to appreciate from the raster plots – temporal structure is not obvious. Consider adding PSTHs for each stimulus condition to substantiate this claim.

Done.

4. Figure legends, P31, Figure 1 references a ‘t-SNE’ plot. Although directly Google-able, this term/type of plot may be sufficiently unfamiliar that a sub-sentence explaining what is shown may be warranted.

Done.

Reviewer #2: Review of Horwitz, "Temporal information ..."

This paper presents measurements of single unit responses in primate LGN, made for peri-threshold stimuli, and analysis of the data that relate the information about achromatic stimuli carried by the LGN neurons to behavioral threshold and to the information available at earlier stages in visual processing. It shows the the dominant locus of loss of high temporal frequency information in the visual system is early, with most of the loss occurring because of temporal filtering in the cones. This is an important result, because the cones are so early in the processing chain that the implications of the loss apply to essentially any visual stimulus and task.

The paper is nicely situated within a broader enterprise whose goal is to understand what visual information makes it through the retina to the cortex - understanding the information loss in early vision is important because it delineates the information processing problem the cortex must solve. An enduring lesson from analyses of this sort is that the effect of early vision upon performance can have large effects that generalize well across a wide range of stimulus and task conditions.

Overall, the paper describes a substantial body of careful experimental and computational work that brings together data and theory to speak to fundamental questions in visual information processing.  I think publication in PLoS Comp Bio will be appropriate, after the paper is revised to address the points below.

BIG PICTURE

* The introduction might do a bit more of expressing the author's take on why the broad line of work should be of broad interest to sensory neuroscientists, and why addressing this question in the context of understanding thresholds is a strong approach.

Done. I hope the reviewer does not mind my using some of their language in this change.

* The paper might do a bit more work in the introduction and discussion to emphasize why running the neural experiments near threshold is important. The approach reduces the dependence of conclusions on extrapolations from supra-threshold measurements to peri-threshold SNR, and this should be articulated more clearly. The method is one of the best if one wants to link SNR of neural representations to behavior. The intro should say why we care about the answer to this question as well.  Note that where I found the statement "Critically for the logic of this experiment ..." was on page 15 of the methods.

Done.

* The paper acknowledges in various places that efficiency of the cone and neural codes relative to behavior depends rather critically on stimulus duration. This is also true of spatial extent, although that variable is not manipulated in this study.  While behavioral threshold will more or less asymptote as stimulus size and duration are increased, neural information will continue to increase. For present purposes, as noted towards the end, efficiency as a function of TF depends on whether one arranges the stimuli to match behavioral integration time separately across each TF, or computes it with respect to constant duration.  Of particular importance is that it isn't necessarily mysterious that neural efficiencies could exceed behavioral efficiencies, if the neural data are collected for long integration times. I think this point needs to be made more clearly earlier on, and that it would be helpful to analyze the data by using a constant cycles integration time that is a good match to estimates of behavior - that would help us understand how much of the tf dependence can be accounted for by the change in integration time known to occur behaviorally.  The analysis could be done by extracting the first part of each response, as is already done in a side analysis but not taken all the way to efficiency.

I agree that there is nothing mysterious about neural efficiencies exceeding behavioral efficiencies given long integration times and include a brief, frank statement on this issue in the revised Introduction.

I am including a figure (Supporting Figure 2) that shows the results of an analysis of individual response cycles (taken from the middle of the stimulus presentation duration, when LGN responses are strongest). When the ideal observer has access to only a single cycle of the LGN response, signal-to-noise is fairly flat across temporal frequency and reasonably well-matched to behavior for magnocellular neurons. This is an expected consequence of the increase in signal-to-noise ratio with temporal frequency in fixed integration windows and the decrease in stimulus period with temporal frequency.

At some point in the visual system, integrating for longer epochs presumably does not continue to increase SNR. Inefficient behavioral integration is implemented by neurons that also integrate inefficiently. Finding representations in the early visual system with SNR that approaches the SNR of these decision-related representations is a goal of this research program.

* There are lot of numbers and estimates that go into the modeling. I think it would be useful for others who want to build on this work if there were a table of parameters, what numerical values were used for each, and the source of the estimates.  A thoughtful comment about reasonable bounds on the estimates would also be valuable.

I have added such a table to the manuscript. Access to the code here should help here as well. Please see https://github.com/horwitzlab/LGN-temporal-contrast-sensitivity for a repository containing the LGN data and the Matlab code for the ideal observer analyses.

* Data availability. The header info indicates under data availability: "Yes - all data are fully available without restriction"  My understanding is that PLOS requires that all data be provided in a curated archive (e.g. fig share) - I do not regard a statement that the data may be obtained from the author upon request as sufficient to meet the journal's state standard (from info for authors): "For all submissions, authors must clearly provide detail, data, and software to ensure readers' ability to reproduce the models, methods, and results."  I would believe that the modeling code should be made available in some accessible way. It would be good to be able to examime these repositories as part of evaluating a revision.  The editor may want to provide additional guidance here about what does and does not satisfy journal policy.

Please see https://github.com/horwitzlab/monkey-temporal-contrast-sensitivity for data and code that is the basis for the behavioral model used in these experiments and https://github.com/horwitzlab/LGN-temporal-contrast-sensitivity for the LGN data and the code for the ideal observer analyses

SPECIFIC COMMENTS

Page 3. The idea in the last sentence of this page, which is explained in Results, is very hard to grasp here.  May not be needed as part of the introduction, especially as there are presumably other plausible mechanisms.

Deleted.

Figure 1. What is t-SNE?  What are the axes in the plot?  How much of the variance did the first nine basis vectors capture?

A reference for t-distributed stochastic neighbor embedding (t-SNE) is provided in the revised figure legend. The axes lack a useful interpretation. The singular value decomposition preserved an average of 78±10% (SD) of the variance in the STAs across neurons.

Figure 2. To fully understand the data plotted here, it is critical to know how contrast is varying with temporal frequency for these neurons. As I understand it, the answer will depend on the precise eccentricity of each neuron.  Since within neuron, contrast covaries perfectly with temporal frequency, the contrast information could be plotted as a function of TF, with contrast scale given as a right vertical axis.  Also, what do the shaded regions represent - presumably STD or SEM.  Presumably, threshold contrast was matched separately to the eccentricity of each neuron, this should be stated explicitly. In general, the figure legends are rather sparse and a careful read to make sure that what each type of point shows is explained clearly in each figure legend would be helpful.

The suggested changes to Figure 2 have been made. The shaded regions represent bootstrap estimates of the standard error of d'. All figure legends have been revised to explain each part of each figure better.

Figure 3. Is there enough data to add to this figure some indication of whether there are interactions between eccentricity and temporal frequency?

The data are not sufficiently strong to make strong case for or against such an interaction (see Reviewer Figure 1). The d' vs. temporal frequency tuning curves looked qualitatively similar across the eccentricities I tested.

One can more or less infer that in C and D, the circles are monkey 1 and triangles monkey 2.  And one can also guess that closed is parvo and open is magno.  But why make us work so hard? Please say this sort of thing explicitly in every legend.

Done.

Figure 4. There are presumably error bars on behavioral d', which should be shown in plots like this one.

This is challenging, and I have done my best to address this point in the revised manuscript. The behavioral threshold model provides an estimate of the contrast that yields 82% correct in the 2AFC task (which varies as a function of retinal position, color, and temporal frequency). Under the model, errors are homoskedastic, so I pooled data across retinal position, color, and temporal frequency to estimate a single scalar (cross-validated) prediction error in % contrast (feeling slightly queasy). I then mapped these prediction errors onto percent correct (and thus, d'), by assuming a Weibull psychometric function with a slope of 3 (and feeling a little queasier). The interval that comes out the other side, [d': 0.89 1.70], does not have a rigorous interpretation as far as I know, but it provides a qualitative sense of how variable the behavioral d' estimate is. Alternative suggestions are welcome.

As with Figure 3, interpretation of this figure requires understanding whether there are interactions between temporal frequency and efficiency.  I also think it is worth reminding readers when this figure is discussed that the absolute relation between behavioral and neural d' will vary for well-understood reasons on stimulus duration and size.

Done.

Page 5. "If monkeys were ideal ...." Because this statement follows right on an analysis of individual neuron d', the reader has in mind that the statement refers to the relation between what is shown in Figure 4 for neural snr (d') and behavior.  But the statement applies to the next case, where the population information is considered.  I would move this down to after Figure 5 is introduced.  (I do understand that if the average neuron d' has an tf dependence, as in Figure 4, it will be hard for the population d' not to. But this takes some thinking.  If the passage in question is kept where it is, the logic should be unpacked more.  Also note that Figure 4 averages across a lot of eccentricities, and depending on exactly what happens at each eccentricity wrt tf dependence, and how the neurons are sampled across eccentricity, things do get a little complicated to unpack.)

Done.

Page 6. Another reason the LGN pop info might exceed cone current info, as calculated, is that there is more correlation between responses of LGN units than assumed.  Could list that here.

This is a good idea, but I think the problem is more likely to lie in the assumptions of LGN RF size or in the SNR of currents in single cones at high temporal frequencies. As shown in Supporting Figure 1, even *individual* LGN neurons from monkey 2 appear to have greater d' than then the cones inside their RFs at high temporal frequencies.

The correlations between LGN neurons within a mosaic are assumed to be equal to their RF overlap. Thus, these interneuronal correlations compensate for the "double counting" of contrast signals that would otherwise occur at these regions of overlap. Reducing RF overlap therefore reduces interneuronal correlations without changing population SNR by much. For example, truncating the Gaussian RFs at 4 standard deviations (instead of 2) increases the correlation between neighboring neurons by ~30% but decreases the population scale factor by only ~10%.

Figure 6.  It is a little surprising to me that there is no stimulus-dependence to the microsaccade driven histograms.  I think it would be better to show stimulus-present and stimulus-absent separately in any case, and perhaps to comment on why there is no effect.

Done.

Is the SF of the stimulus low enough relative to the size of microsaccades that there is little effect on what each neuron sees across a saccade?

Yes, the median microsaccade amplitude was 0.18°, moving the eyes by only 0.18 stimulus cycles. I was expecting to see a relationship between the direction of microsaccades and the detectability of the 45 Hz stimulus (recall that the stimulus always drifted upward, and a microsaccade lasts ~20 ms which is close to the stimulus period) but I failed to find this effect in the data.

The stereotyped activity modulation time-locked to microsaccades (Figure 6E–6L) probably swamps the effects of the stimulus on the response at the single neuron level.

Figure 7.  I think from the main text that two durations are shown here, with red being 666 ms and black being 333 msec.  But the legend should make it clear.

Done.

Page 7. "SNR in parvocellular ... result is consistent with high levels of noise ..." I think the point being made is that the data show that result is not a result of high levels of noise, so might reword to make that the lead, rather than first saying result is consistent with an idea and then demonstrating that the conjecture was incorrect.

Done.

Page 8. I know I'm a broken record on this point, but this is way too late to be telling us about the fact that behavioral integration time is known to vary with TF. And we should also know early on that behavioral integration time is known to be considerably less than the 666 msec used in the recordings. These two observations are key to understanding all of the results and need to come earlier.

Done.

Page 8. The constant spike model is interesting enough, but isn't tested in any way that I see by these experiments. I think it is fine to list it, but can other ways neural mechanisms might cause tf dependent integration times be listed?  Or is this the only idea in town?

The data constrain the mechanisms by which LGN signals are read out to inform psychophysical decisions but not strongly. The analysis shows that temporal frequency has a monotonic relationship to several easy-to-calculate statistics of LGN spike trains (e.g. counts, rates, interspike-intervals, and modulation amplitudes), any of which might be linked to read-out efficiency. Thinking about the stimulus in terms of its representation at the level of LGN suggests a family of mechanisms that might explain the dependence of temporal integration on frequency (a dependence which is more mysterious if one thinks only about the stimulus on the retina). This is clarified in the revised text.

Page 9. At 1 cpd, I doubt fixational drift (as opposed to microsaccades) matters,

but perhaps this should be stated expliclity.

Done.

Page 10. "No model ..." I agree that we don't have a model that we're sure is correct.  But it's not quite as hopeless as the author makes it sound here.  Calculations of the sort the author presents could be used to produce stimuli that are constant d' at the cone mosaic, and these would make the basis of a useful experiment.  Might discuss.  Also, Watson's window of visibility model does, I think, predict the relevant thresholds.  Perhaps the model is not right, but it is certainly worth a mention.

Done.

Page 11. "Shorter counting windows ... (Figure 10)"  I am not following the point here, particularly in terms of any relation to Figure 10.

This paragraph has been deleted.

Page 12. Note also that if the spatial size of the stimulus exceeds the spatial integration area of the visual system, that will also make the neural information look larger than it otherwise would with respect to behavioral performance.  Moreover, because the temporal properties of neural centers and surrounds can differ, it is not guaranteed that the way the information transmission behaves with temporal frequency is independent of spatial size of the stimuli.  This should be discussed where the non-separability of neural responses is mentioned.

Done.

Page 12. d' from LGN exceeded upper bound. Time to mention spatial and temporal integration areas/durations again.

Done.

Page 14. The discussion just sort of fades out.  I think a short concluding paragraph summarizing the key conclusions here would help.

Done.

Page 16. Say explicitly that the stimulus modulations were isochromatic, and thus seen by the S cones, but that S cones are not thought to contribute much if anything to detection of isochromatic gratings because they do not contribute to the luminance mechanism. Or if I misunderstand, and the modulations drove only the L and M cones (and thus had a luminance and chromatic component), that too should be stated.

The stimulus did not modulate the S-cones, but under most conditions it did not appear colorful to me. Recall that it was small, parafoveal, and usually drifting quickly. I had ample exposure to it (I am subject H2 in Gelfand and Horwitz, 2018).

Page 17.  Isn't photocurrent noise spectrum temporal frequency dependent? If so, the analysis used to get d' from photocurrent seems wrong, as it is based on a mean difference in response, as described. At minimum, add more detail to be clear.  And if tf dependence of noise spectrum is not being accounted for, either redo analysis so that it is, or explain why it need not be.

The reviewer is correct that the photocurrent noise spectrum is temporal frequency-dependent. The ideal observer of cone currents takes this fact into account, and this was inadequately explained. The stochastic component was represented in the Fourier domain as the square root of the noise power spectrum, where it was multiplied by the Fourier transform of the spatiotemporal weighting function. Consequently, noise components were weighted by their presence in the signal. This weighted noise amplitude spectrum was then squared, summed, and divided by N (the number of Fourier components) to calculate the variance of the cone current noise (by Plancherel's theorem). Code implementing these calculations can be found at https://github.com/horwitzlab/LGN-temporal-contrast-sensitivity/IsoSampConeCurrent.m

Strictly speaking, the ideal observer should weight frequencies according to signal-to-noise ratio, not the signal alone, as we have done. The difference ends up being negligible. For a given stimulus, the noise amplitude is nearly constant across the frequencies at which any given stimulus has appreciable power.

Page 19, eq 9. Is it OK to approximate with a single covariance matrix, common across signal present and signal absent?

The covariance matrix is not generally the same on signal-present and signal-absent trials. Ideally, I would characterize this dependence and use it to discriminate signal-present from signal-absent trials, but this would require additional assumptions about (or estimates of) response phase. Instead, I've chosen the simpler route of calculating a statistic, (*"dist"* in Equation 10 of the manuscript), that (a) has an approximately N(0,1) distribution under the null hypothesis of an unmodulated spike train and (b) increases monotonically with the type of departures from the null hypothesis we observe in the data (modulations of firing rate at the fundamental frequency of the stimulus). Empirically, *dist* has an approximately N(μ,1) distribution in many stimulus present conditions, and I am happy to provide documentation on this if the reviewer thinks this would be useful.

Page 21, eq 12. It might be nice to unpack this interesting formula a little.  For example, the number of neurons is implicit in the vector lengths. Does the formula scale intuitively as one adds in iid neurons?  That sort of thing.

Done.

Reviewer #3: In this paper, Horwitz explores the loci of information loss in the macaque visual system using a combination of computational modeling, electrophysiological recordings from the LGN and perceptual reports.

This is an important question in systems neuroscience and the paper is overall well written. A weakness of the paper is the fact that some of the key results of the paper hinge on model assumptions about photon transductions and electrical currents in cones, and on population scaling factors of LGN neurons. The model assumptions seem reasonable; however, I wish the author had explored and illustrated how the results would have changed over a range of model parameters.

A table of parameter values, sources, and previous estimates is provided in the revised manuscript.

Matlab code implementing the photon counting and cone current ideal observers is available at https://github.com/horwitzlab/LGN-temporal-contrast-sensitivity. Both ideal observers are based on assumptions about cone density as a function of retinal eccentricity, axial cone collecting area, and the absorption of light in ocular media. All of these assumptions were matched for the two ideal observers.

The LGN pooling model is based on assumptions about RF size, RF overlap, noise correlation within ON and OFF mosaics, and noise correlation between ON and OFF mosaics. The function IsoSampGetPopulationScaleFactor.m (available from https://github.com/horwitzlab/LGN-temporal-contrast-sensitivity) allows users to calculate population scale factors for arbitrary RF sizes and interneuronal correlations. The revised Discussion and Supporting Figure 3 provide results of some of these manipulations.

The introduction is very sparse and reads more like a summary of results.

This has been corrected.

Please describe the behavioral experiment in the Methods section.

Done.
