# Supplementary material for: Temporal information loss in the macaque early visual system
Source: PLoS Biol. 2020 Jan 23;18(1):e3000570. doi: 10.1371/journal.pbio.3000570 (PMC6977937; doi:10.1371/journal.pbio.3000570)
Supplement: Attachment — Submitted filename: ResponseToReviewers.docx [file pbio.3000570.s008.docx]

-- Please include an ID number of the protocol(s) approved by the University of Washington Institutional Animal Care and Use Committee.

Done.

Regardless of the method selected, please ensure that you provide the individual numerical values that underlie the summary data displayed in the following figures: 1, 2, 3, 4, 5, 6, 7, 8, S1, S2, S3, S4, and S5.

The raw data have been uploaded to <https://github.com/horwitzlab>.

Please also ensure that the figure legends in your manuscript include information on where the underlying data can be found, and ensure your supplemental data file/s has a legend.

Done.

Please ensure that your Data Statement in the submission system accurately describes where your data can be found.

Done.

This study uses Ideal Observer-type models to assess loss of information at the level of the photoreceptors and LGN. Previous work assessed in detail the loss of visual information across the retina, from the photoreceptors to the ganglion cells, also using Ideal Observer type model analysis (Borghuis et al., 2009, J Neurosci. 2009, 'Loss of sensitivity in an analog neural circuit'; currently not cited in the manuscript). This study should be cited in Introduction, and it should be made clear in Discussion how the current results relate to those of the prior study. For example, while the prior study did not assess loss across frequency bands, it appears that claims about the origin and magnitude of losses (for example, within the photoreceptors based on assumed quantum efficiency as reported) can, and should be, compared.

Borghuis et al. 2009 is cited in the revised Introduction and Discussion. Both the current study and that of Borghuis et al. 2009 found that most of the SNR loss for visual contrast detection occurs in the retina. This consistency of results, despite different model systems and ideal observer models, is emphasized in the revised Discussion.

My one suggestion is that perhaps the discussion could benefit from a simple summary figure that divided up the information loss across the three stages (photoisomerizations to photocurrent, photocurrent to LGN, LGN to behavior) as a function of temporal frequency.  Such a plot would allow easy grasp of the main messages of the paper.  Figure 5 provides this type of information, but perhaps in the discussion a version that expressed the result in terms of percentage information loss, that averaged across the two monkeys, and that combined the information carried by parvo/magno into one single plot would be helpful to go along with the words.

A final figure has been added to the Discussion to illustrates the percentage information loss across stages of the visual system as a function of temporal frequency.
